# Insights into the Role of Histone Methylation in Brain Aging and Potential Therapeutic Interventions

**DOI:** 10.3390/ijms242417339

**Published:** 2023-12-11

**Authors:** Nikolaos Vitorakis, Christina Piperi

**Affiliations:** Department of Biological Chemistry, Medical School, National and Kapodistrian University of Athens, 75 M. Asias Street, 11527 Athens, Greece; smd2100015@uoa.gr

**Keywords:** histones, methylation, aging, brain, histone marks, H3K4me3, H3K27me3, H3K9me3, H3K36me3, UNC0042, diet restriction

## Abstract

Epigenetic mechanisms play a primary role in the cellular damage associated with brain aging. Histone posttranslational modifications represent intrinsic molecular alterations essential for proper physiological functioning, while divergent expression and activity have been detected in several aspects of brain aging. Aberrant histone methylation has been involved in neural stem cell (NSC) quiescence, microglial deficits, inflammatory processes, memory impairment, cognitive decline, neurodegenerative diseases, and schizophrenia. Herein, we provide an overview of recent studies on epigenetic regulation of brain tissue aging, mainly focusing on the role of histone methylation in different cellular and functional aspects of the aging process. Emerging targeting strategies of histone methylation are further explored, including neuroprotective drugs, natural compounds, and lifestyle modifications with therapeutic potential towards the aging process of the brain.

## 1. Introduction

Aging is defined as the progressive decline of functional integrity that leads to impaired function and increased vulnerability to death. In turn, the aging of brain tissue is characterized by shrinking in volume and changes at the molecular level and morphology [1]. The rate of neuronal death varies according to specific brain regions, with the most notable changes taking place in the frontal and temporal cortex, thalamus, putamen, and nucleus accumbens. In other regions, such as the hippocampus, the number of dying neuronal cells is compensated by the generation of new ones by the neural stem cells [2].

Generally, aging affects the brain both microscopically and macroscopically. At the cellular level, the aging process has been shown to induce shrinkage of neuron cells, deterioration of dendritic cells, deceleration of cell metabolism, activation of microglia, induction of white matter lesions, as well as demyelination and small vessel disease. Macroscopically, the brain’s phenotype is also affected, exhibiting a reduction in brain size, thinning of the cortex, diminished gyral complexity, deterioration of white matter, and enlargement of the ventricles [3]. All these events lead to cognitive impairment, affecting memory, attention, and processing speed.

Brain aging takes place in every human during their lifetime, and although it cannot be classified as a disease, it exhibits several similarities, including structural, functional, and biochemical changes that compromise proper functioning and cause several symptoms [4]. Moreover, brain aging serves as a risk factor for many diseases, creating a favorable environment for their progression through the accumulation of damaged proteins, oxidative stress, and inflammation in combination with the declining efficiency of the brain’s detoxifying mechanisms.

Diseases that have been associated with brain aging include Alzheimer’s (AD) and Parkinson’s disease (PD), Amyotrophic lateral sclerosis (ALS), multiple sclerosis (MS), vascular and Frontotemporal Dementia (FTD), as well as Lewy body dementia and Huntington’s disease (HD). AD is a neurodegenerative disorder that predominantly affects the elderly population and is marked by the buildup of atypical protein formations such as tau tangles and amyloid plaques in the brain, which result in cognitive deterioration, memory impairment, and changes in behavior. Cross-sectional studies involving healthy elderly individuals, patients with mild cognitive impairment, or AD, have demonstrated that neurodegenerative diseases expedite the aging process and induce significant structural transformations within the brain [5].

Parkinson’s disease is another neurodegenerative disease related to brain aging that affects movement control. It is defined by the depletion of neurons in the brain responsible for producing dopamine, resulting in motor issues such as tremors, stiffness, and slowness of movement. It may often lead to non-motor symptoms like cognitive deficits and mood disorders [5]. Furthermore, ALS is marked by the degeneration or impaired function of motor neurons in the central nervous system (CNS) and progresses during brain aging. ALS results in the shrinkage and gradual weakening of voluntary skeletal muscles [6].

Multiple sclerosis is characterized by a significant physical disability that is not trauma-related and is also linked to the brain aging process. This condition is characterized by the loss of neurons and distinct neuroinflammatory demyelinating lesions. Moreover, vascular dementia is a condition distinguished by diminished blood circulation to the brain as a result of aging, which leads to alterations in blood vessels, causing ischemic episodes in different brain regions. In addition, Frontotemporal Dementia (FTD) comprises a collection of rare neurodegenerative diseases that predominantly impact the temporal and frontal lobes of the brain. FTD may lead to alterations in behavior and language, usually affecting people earlier in life in contrast to AD. Mild Cognitive Impairment (MCI), an additional brain aging-related disorder, involves changes in cognition that are more substantial than the ones in typical age-related cognitive decline but less than the ones in dementia. MCI can serve as a precursor to severe conditions like Alzheimer’s disease. Furthermore, Lewy body dementia is a progressive brain disorder marked by the existence of anomalous protein deposits in nerve cells, called Lewy bodies. This condition can lead to a plethora of motor, cognitive, and psychiatric symptoms, such as hallucinations and variations in the levels of alertness. Additionally, Huntington’s disease is a genetic disorder characterized by the progressive degeneration of brain cells, resulting in motor symptoms, psychiatric disturbances, and cognitive decline. It is therefore evident that elucidation of the molecular mechanisms that contribute to brain aging is of primary importance [5].

Epigenetic modifications have been proposed to underline the process of brain aging through regulation of metabolic pathways by activating or repressing gene function. Epigenetics provides the link between environment and gene expression according to the requirements of the organism and depends both on endogenous and exogenous factors such as synaptic alterations, hormonal changes, dietary factors, exercise, and stress, respectively. Gene expression regulation is dynamic and can be affected at different stages of development by a variety of stimuli.

Among the main epigenetic mechanisms, DNA methylation, histone modifications, and noncoding RNAs are the most prominent. In turn, histone methylation is the most enduring and stable posttranslational modification, as well as a well-studied mechanism of gene silencing and repression [7]. However, histone methylation may also promote transcription activation, depending on the specific amino acid modified [8].

In this review, we specifically explore the impact of histone methylation as a contributing factor to brain aging. We have performed a detailed search of the PubMed database for peer-reviewed articles investigating the epigenetic mechanisms underlying brain aging, published in the English language during the last ten years. We used the terms “brain aging”, “epigenetics”, “histone methylation”, “neurodegenerative disorder”, “neurogenesis”, “cognitive decline”, “neurobiology of aging”, and “brain health” while also using Boolean operators to combine them effectively.

In the following sections, we provide an overview of the basic aspects of epigenetic mechanisms, with a focus on histone posttranslational modifications. We particularly explore prominent histone methylation patterns found in the normal brain, their susceptibility to age-related alterations, their relevance to diseases, and their potential as targets of therapeutic intervention.

## 2. Basic Aspects of Epigenetic Mechanisms

Epigenetics refers to the study of changes in gene function that are mitotically and/or meiotically inherited without alterations in DNA sequence. These chemical changes are reversible and can be mediated by three main mechanisms, including DNA methylation, histone posttranslational modifications of N-terminal amino acids, and non-coding RNA.

Covalent modifications of DNA mainly involve methylation of cytosine residues, but guanine and adenine can also be modified [9]. Methylation of cytosines takes place primarily within CpG dinucleotides, resulting in the formation of 5-methylcytosine (5-mC), but it may also occur in non-CpG sequences. Specific enzymes, namely DNA methyltransferases (DNMTs), catalyze the transfer of methyl groups to DNA from S-adenosyl-L-methionine (AdoMet). DNA methylation in the promoter region can suppress gene transcription either by preventing the binding of transcription factors or by encouraging the binding of transcriptional repressors. CpG methylation is critically involved in processes such as X-chromosome inactivation, imprinting, suppression of transposons and repetitive elements, regulation of tissue-specific gene expression during development and differentiation, as well as various other functions. The DNA methylating enzyme DNMT1 mediates the mitotic inheritance of DNA methylation sites, while DNMT3A and DNMT3B catalyze the de novo methylation of unmethylated bases, depending on cell type and developmental stage. The control of gene expression in embryonic stem cells can also be achieved through the methylation of cytosines outside of CpG sequences. At the same time, DNA demethylation is a less understood subject in today’s medicine. The ten-eleven translocation (TET) enzymes’ oxidation of 5-methylcytosines (5mCs) to hydroxymethyl cytosines is a demethylation mechanism, with TET2 being the main enzyme [10].

Age-related declines in DNMT1 expression lead to lower levels of DNA methylation. The expression of DNMT3A and DNMT3B, on the other hand, rises with age, contributing to de novo methylation of CpG islands. When it comes to TET2, the study of Buscarlet et al., recently showed that age-related TET2 mutations may lead to a decrease of 5-mC in human blood cells [11,12].

Other epigenetic regulators, the non-coding RNAs (ncRNAs), have received increasing attention due to their implication in intracellular signaling and gene activity. They encompass several categories, such as microRNAs (miRNAs) and small interfering RNAs (siRNAs), and may exert both an activating and suppressive role in gene function [13]. LncRNAs can also interact with RNA-binding proteins, leading to histone deacetylation or hindering transcription factor binding at promoter regions. Consequently, lncRNAs are integral to processes like imprinting, X-chromosome inactivation, and cardiac development. Small non-coding RNAs (sncRNAs), including miRNAs and siRNAs, influence transcriptional gene silencing through epigenetic modifications. The initiation of transcription can be hindered by some small interfering RNAs (siRNAs), which do not cause epigenetic alterations to histones and DNA. ncRNAs play a vital role in maintaining epigenetic changes during differentiation, contributing to the regulation of necessary gene expression patterns [14].

Histones, on the other hand, are extremely basic proteins that comprise the building blocks of nucleosomes, ensuring proper organization of chromatin structure and gene transcription regulation. Double copies of the four main histones, namely H1, H2A, H2B, H3, and H4, compose the histone octamer, the fundamental unit of nucleosomes that enables proper wrapping of double-stranded DNA. A large variety of histone-modifying enzymes can induce multiple posttranslational modifications on specific amino acid residues (lysine, serine, and arginine) present on histones’ amino-terminal tails. Different types of modifications, such as acetylation, methylation, phosphorylation, ubiquitination, and sumoylation, can take place, which can modulate gene transcription by either promoting or restricting gene expression [15].

The significant correlation of specific posttranslational histone modifications with transcriptional events is of primary importance and is discussed in more detail in the following sections.

## 3. Impact of Histone Modifications in Gene Regulation

Histone acetylation is a central mechanism of gene activation involving the addition of acetyl groups to specific lysine residues in histone protein tails. This reaction is mediated by specific enzymes known as histone acetyltransferases (HATs), which employ the cofactor acetyl CoA to add the acetyl group to the ε-amino group of lysine residues. Acetylation loosens the interaction between the negatively charged DNA and the positively charged lysine, opening the chromatin structure and enabling genes’ accessibility to transcriptional machinery [16]. It serves as a switch that allows interconversion between permissive and repressive chromatin structures [17].

The opposite reaction which is the removal of acetyl groups from histone terminals, has a repressive impact on gene regulation, inducing the compaction of chromatin and diminishing its accessibility. It is mediated by histone deacetylases (HDACs), which catalyze the removal of acetyl functional groups from histone and non-histone proteins. They regulate deacetylation in groups with other HDACs or other enzymes and have low substrate specificity. The equilibrium between HAT and HDAC activities constitutes a fundamental regulatory mechanism for gene expression, affecting various facets of cellular function [16].

The histone methylation process involves the addition of methyl groups (–CH3) to the ε-amino group of lysine and arginine residues of histone proteins. Methylation of histones can either silence genes in heterochromatin areas or modulate gene expression at euchromatic loci, indicating its versatility in regulating transcription. This process is catalyzed by histone methyltransferases (HMTs), which utilize S-adenosyl-methionine (SAM) for methyl groups and add them to lysine and arginine residues located on the tails of histones [18].

The opposite function of methyl group removal from lysine and arginine residues of histones is mediated by demethylases [19]. The significance of histone methylation becomes particularly evident in the context of the DNA damage response (DDR), where numerous methyltransferases and demethylases accumulate in damaged DNA loci in order to alter chromatin and mediate the respective response to DNA damage [18].

Dysregulation and genomic lesions affecting the expression and activity of methyltransferases and demethylases have been implicated in cancer and neurological diseases, underscoring the pivotal role of histone methylation in health and disease [20,21].

Histone phosphorylation is implicated in transcriptional activation through the addition of phosphate groups to serine and tyrosine residues. This process is catalyzed by kinases, while the opposite reaction is catalyzed by phosphatases. Phosphorylated histones are involved in transcriptional activities, including the regulation of chromatin compaction during processes like mitosis, meiosis, and apoptosis, DNA damage repair, and aging [22]. Histone phosphorylation works in conjunction with other histone modifications and establishes a platform for mutual interactions involved in chromatin remodeling [23].

Histone ubiquitination involves the covalent attachment of ubiquitin, a 76-amino acid protein, to other protein targets through the ubiquitin-proteasome system to control the stability and degradation of the substrate protein. This process is facilitated by histone ubiquitin ligases and can be reversed by deubiquitinating enzymes (DUBs) [24]. Mono-ubiquitination is essential for transcriptional regulation, DNA damage signaling, and protein translocation, while polyubiquitination serves as a tagging mechanism for proteins, marking them for degradation or activation through specific signaling pathways [25]. Histone ubiquitination’s main function is the DDR involvement, where it can mediate rapid chromatin responses, facilitating the availability of a variety of histone posttranslational modifications to respond to and resolve potentially dangerous conditions [26].

## 4. Histone Methylation Alterations Involved in Brain Aging

Genetic factors have long been recognized as important contributors to brain aging, but emerging research has also revealed the crucial role of epigenetic modifications, particularly a decline in histone acetylation and a rise in histone methylation, in shaping the aging brain. Epigenetic marks in the brain change during development and also during the aging process [5,13,15]. Loss of histone acetylation is a key determinant of aging-mediated chromatin remodeling, leading to compaction and repression of genes associated with brain development and protection, as well as synaptic plasticity and memory formation. As histone acetylation and deacetylation balance is progressively disrupted during aging, an elevation of histone methylation is observed with a dual role in gene activation and repression, further affecting brain function and memory formation [27].

Histone methylation patterns within the normal brain exhibit remarkable complexity and context-specificity. Firstly, histone 3 lysine 4 trimethylation (H3K4me3) is a histone mark established by MLL2 (KMT2B), SMYD3, and SET1A methyltransferases. It is mainly associated with the activation of gene transcription, located at the promoters of genes of the neuronal lineage such as *Otx1*, *Fam72a*, *Bahcc1*, and *2610017I09Rik*, which are involved in the plasticity of the synapses, neuronal differentiation, and the formation of memories [28]. In the human prefrontal cortex of aged individuals (>60 years), there is a decrease in the H3K4me3 mark at 556 genes that are mainly involved in neuronal development, such as the neurogenesis transcription factors *NEUROD1*, *NEUROD6*, *NEUROG2*, and members of the semaphorin and cadherin adhesion molecules *SEMA3A*, *-3F*, *-6A*, and *CDH3*, *-9*, which are involved in neuronal growth, differentiation, and connectivity. At the same time, an increase of 101 genes was detected in the brains of aged individuals compared to the brains of young ones (<1 year) [29], which was shown to impair transcriptional activity and affect vital neuronal functions. It is therefore evident that significant remodeling of the H3K4me3 mark takes place with brain aging in a way that relies upon a specific condition, background, or environment for proper interpretation.

Another common histone methylation mark is H3K27me3, associated with gene silencing. This histone mark is established by Enhancer of zeste homolog 2 (EZH2), the histone methyltransferase of the Polycomb Repressive Complex 2 (PRC2), and has been detected in genes involved in cell differentiation, synaptic function, neurodegeneration, and epilepsy, as well as in maintaining the distinct characteristics and functions of various cell types within an organism or tissue, such as *NEUROG2*, *OLIG2*, and *HOXA1-5* [30,31]. Studies in SAMP8 (Senescence Accelerated Mouse-Prone 8) mice, which exhibit an expedited aging phenotype characterized by neurodegeneration and cognitive impairment, have demonstrated that there is a clear increase in this mark during aging [32]. This elevation leads to a dysregulation of normal neuronal function, which is a typical cause of brain aging advancement. Furthermore, studies have shown that broad genome regions are enriched for H3K4me3 and H3K27me3 marks in senescent cells but not in proliferating cells. These regions are highly associated with the upregulation of genes involved in the senescence-associated secretory phenotype (SASP) and indicate a broad redistribution of these marks during aging [32].

H3K9me3 is one more repressive histone mark found at repetitive DNA sequences and transposons, established by the SET domain-containing H3K9 methyltransferases (SUV39H1, SETDB1). It is required at the early stages of DDR and for the maintenance of genomic stability in neurons. This mark has been shown to increase with aging in *Drosophila* and humans [32]. Moreover, dysregulation of H3K9me3 in the brain has been associated with aberrant gene expression and neurological disorders. Elevated H3K9me3 induces irregular heterochromatin remodeling, which causes suppression of genes involved in synaptic function such as *Bdnf*, *SNAP29*, *RABs (3A, 14)*, *SEC22 (A*, *B*), *syntaxin 6*, *synaptobrevin-like 1*, *dynamin-1*, and *dynamin-like 1* [33,34]. In this way, altered H3K9me3 levels have been associated with synaptic dysfunctions that worsen sporadic AD.

Lastly, the histone mark H3K36me3 is often present in the bodies of actively transcribed genes, facilitating efficient transcriptional elongation and being involved in DNA damage response. This histone mark is established by the SET domain containing 2 SETD2 and -5 (SETD5) methyltransferases and has been shown to regulate genes involved in neuronal development and differentiation, DNA methylation, maintenance of genomic stability after damage, and alternative splicing [35]. In vivo experiments in SAMP8 mice and *Drosophila* demonstrate reduced levels of this mark in their brains, indicating low DNA repair efficiency [32]. Moreover, H3K36me3 is required for DNMT3B binding and activity, while reduced expression of this mark has been involved in specific genes repression such as *Bdnf* and *Gfap* [36]. However, the reduced H3K27ac levels due to increased HDAC2 activity have also been involved in the downregulation of *Bdnf* gene expression in the hippocampus of older mice [27].

**Figure 1:**
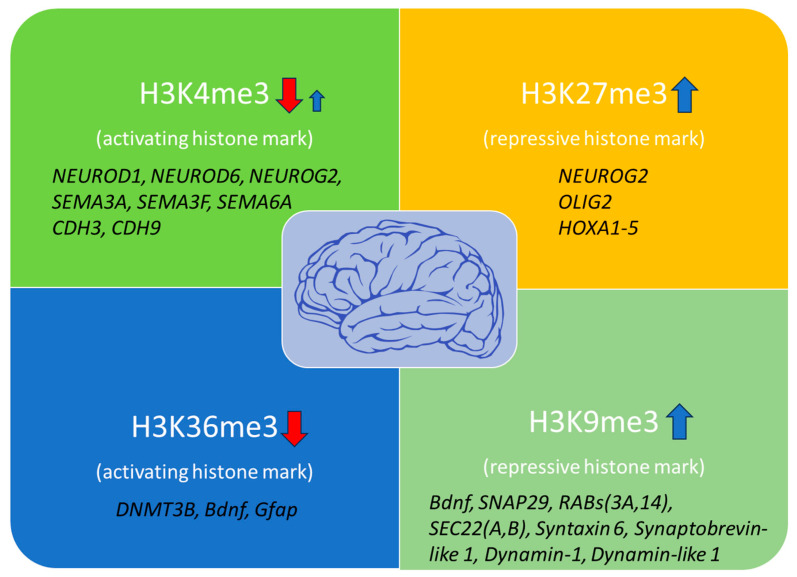
Histone modification marks are associated with brain aging and affected genes. H3K4me3 and H3K36me3 activating marks are mainly decreased with age, impairing transcriptional precision, DNA damage repair, and vital neuronal functions. However, some genes have been shown to exhibit increased H3K4me3 levels, indicating additional gene-specific regulation through this mark during brain aging. At the same time, increased levels of the repressive histone marks H3K27me3 and H3K9me3 have been detected in several genes involved in neuronal differentiation and synaptic plasticity, contributing to overall neuronal dysfunction (red arrows: downregulation; blue arrows: upregulation).

Generally, there is an observed rise in repressive histone marks including H3K9me3, H3K9me2, and H3K27me3, and a decline in activating marks such as H3K4me3 and H3K36me3 in the hippocampus and cerebral cortex of aged animal models [32] (Figure 1).

Single-cell RNA sequencing studies in middle-aged individuals of the hippocampus have revealed that the neural stem cells (NSCs) express lower levels of specific histone lysine demethylases, notably KDM1B, KDM2A, KDM4A, and KDM5D, compared to NSCs from younger individuals [37]. These findings suggest that changes in histone methylation may contribute to the increased quiescence of NSCs during the aging process. In agreement, it has been demonstrated that the expression of KDM1A, which, along with KDM1B, is responsible for demethylating H3K4me1/me2, is essential for the proliferation of NSCs [37]. Furthermore, common H3K4me3 modifications were detected on gene promoters that facilitate quick transitions between activated and quiescent cell states in the early subventricular zone (SVZ). An in vitro study has indicated that aged SVZs neural progenitor cells (NPCs) show distinct clustering based on the intensity of H3K4me3, further supporting the idea that changes in histone modifications might play a role in the imbalance between quiescence and proliferation in aged NSCs [37,38]. Given that H3K4me1/2/3 modifications are primarily related to gene promoters and intragenic start sites of transcription, it is suggested that dysregulation and uncontrolled transcriptional activation could be due to an age-related decline in histone lysine demethylases in NSCs. Nevertheless, additional experiments are needed to empirically investigate this hypothesis [38].

The aging process often aligns with changes in the immune system, which can run in parallel with dysfunction in microglia. Moreover, senescent microglia lose their potential to initiate efficient immune responses and sustain regular synaptic function during aging. This decline contributes to neurodegenerative conditions and cognitive impairment [39]. A recent study has demonstrated a decrease in the levels of Jumonji Domain-Containing Protein 3 (JMJD3), which is responsible for demethylating histone H3K27me3 and activating microglia in the midbrain of aged mice. Reduced levels of JMJD3 in combination with increased H3K27me3 levels further induce inflammatory responses mediated by microglia (Table 1) [40].

Furthermore, aging leads to an increased activity of H3K4 methyltransferase MLL2, causing a buildup of H3K4me2 in the promoter regions of genes associated with stress and diminishing the presence of the histone demethylase JMJD3 (Table 1) [41]. In this way, more proteins associated with stress response are produced, contributing to a decline in cognition while also ending up with increased H3K27me3 and worsening inflammation. Recent data show that stress responses and immune-related inflammation, which increase due to age-related histone methylation alterations, contribute to functional impairment and the degeneration of neurons [42].

**Table 1:** Histone methylation marks and histone-modifying enzymes are implicated in brain aging.

Histone Mark	Expression in Aged Brain	Histone Modifying Enzyme	Function/Model Studied	Target Genes	Reference
**H3K4me3** (activating mark)	Mostly decreased (with some gene-specific increases)	MLL2-HMT	Synaptic plasticity, neuron differentiation, and memory formation in the human cadaver prefrontal cortex	*NEUROD1*, *NEUROD6*, *NEUROG2*, *SEMA3A*, *SEMA3F*, *SEMA6A*, *CDH3*, *CDH9*	[28,29,37,38,42]
**H3K36me3** (activating mark)	Decreased	SETD2, SETD5	Regulation of transcriptional elongation and DNA methylation in *Drosophila* and SAMP8 mice	*DNMT3B*, *Bdnf*, *Gfap*	[27,32,35,36]
**H3K27me3** (repressive mark)	Increased	EZH2-HMT JMJD3-DMT	Maintenance of cell identity in SAMP8 mice	*NEUROG2*, *OLIG2*, *HOXA1-5*	[30,31,32,40,42]
**H3K9me3** (repressive mark)	Increased	EHMT1, GLP, G9a, SETDB1, SUV39H1-HMT KDM4C-DMT	Neuronal genomic stability and rejuvenation of hippocampal memory in *Drosophila* and mice	*Bdnf*, *SNAP29*, *RABs (3A*, *14)*, *SEC22 (A*, *B) syntaxin 6*, *synaptobrevin-like 1*, *dynamin-1*, *dynamin-like 1*	[27,32,33,34]

## 5. Histone Methylation Contribution to Neurodegenerative Diseases, Schizophrenia, Memory Loss, and Cognitive Decline

Age-related alterations in histone modifications could potentially contribute to the reactivation of neuronal cell cycle processes, a common factor in numerous neurodegenerative conditions. It is interesting that increased H3K4me3 has been linked to mature brain cells re-entering the cell cycle, further suggesting that maintaining the proper balance of H3K27me3 is vital for the overall health of brain cells. Insufficient or excessive levels of this modification can disrupt normal gene functioning, interfere with the cell cycle, and contribute to the progression of neurodegenerative diseases [43].

**Figure 2:**
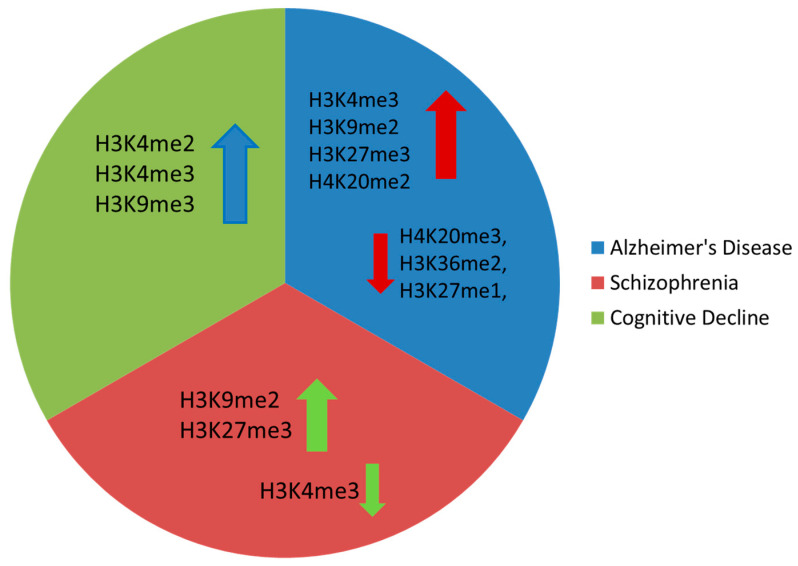
Main histone methylation changes observed in Alzheimer’s disease, schizophrenia, and cognitive decline (arrow up denotes increased levels, arrow down denotes reduced levels).

In this section, we will explore alterations in epigenetic patterns commonly observed in elderly individuals that are associated with the most common neurodegenerative disorder, AD [15]. There is evidence that histone methylation dynamics are dysregulated in AD, with mainly increased H3K9me2, H3K4me3, H3K27me3, H4K20me2, and H3K79me1 levels and decreased H3K79me2, H3K36me2, H4K20me3, H3K27me1, and H3K56me1 marks (Figure 2) [44,45].

In respect to H3K9 levels, a significant rise in H3K9me2 levels and the respective H3K9 methyltransferase euchromatic histone methyltransferase 1 (EHMT1), also known as G9A-like protein (GLP), as well as the epigenetic-related recognition factor, bromodomain adjacent to the zinc finger 2B gene (*BAZ2B*), have been detected in the human prefrontal cortex of aged individuals and AD patients [46,47]. Moreover, the levels of these factors are even higher and associated with disease progression. This observation was also confirmed by the restoration of deficits in recognition, working, and spatial memory through the inhibition of the methyltransferases responsible for this mark [15]. Of importance, G9a, the enzyme that mediates H3K9 dimethylation, has also been associated with cognitive performance in mice [47].

Additionally, the levels of H3K4me3 were found to increase in the prefrontal cortex of a mouse model of tauopathy as well as in AD patients, followed by increased levels of the respective SETD1a/b and MLL1-4 methyltransferases. Of note, the impairment of synaptic function and memory-related behavior associated with these changes was recovered upon selective inhibition of the respective histone methyltransferases mediating this mark [48].

Regarding schizophrenia, there is substantial evidence indicating that it is associated with accelerated brain aging [49]. Aberrant histone methylation has been observed in several psychiatric disorders, and a recent investigation of prefrontal cortex samples of schizophrenia patients showed reduced levels of the activating histone mark H3K4me3 and increased levels of the repressive H3K27me3 mark [49]. Furthermore, a more recent study revealed increased H3K9me2 in the parietal cortex of schizophrenia patients, accompanied by elevated activity of two enzymes, GLP and SETDB1, that catalyze this modification [50]. Additionally, males with schizophrenia were shown to exhibit increased H3K9me2, which was correlated with elevated expression of SETDB1 and G9α methyltransferases. Lastly, genetic variations in the histone lysine demethylase-encoding gene, *KDM4C*, were positively correlated with susceptibility to schizophrenia [51].

The aging process in the brain often results in memory and cognitive deterioration, which have been frequently linked to alterations in the adaptability of dendritic spines’ structure. These changes include a reduction in both the quantity and maturity of spines in aging animals and humans, along with modifications in synaptic communication, further indicating an abnormal neuronal adaptability that directly contributes to compromised brain function [52]. Han et al. detected high H3K4me2 in the promoters of stress-response genes in the prefrontal cortex of aging monkeys, which further resulted in cognition decline due to the overexpression of proteins like tuftelin 1 (TUFT1) and UBX domain protein 4 (UBXN4) [41]. These are involved in adaptation to hypoxia, mesenchymal stem cell function, neurotrophin-nerve growth factor-mediated neuronal differentiation, and ER-associated protein degradation, respectively [53,54]. In agreement, a positive correlation between age and the production of H3K4 methyltransferases was also detected, indicating that an increased stress response linked to H3K4me2 may be caused by increased methylation and not diminished demethylation [41,55]. Brain-Derived Neurotrophic Factor (BDNF), a factor related to brain aging and histone methylation, is also involved in cognitive decline by regulating the adaptability of the hippocampal synapses. Moreover, increased H3K9me3 levels on the *Bdnf* promoter have been observed in the hippocampus of aged mice. Inhibition of SUV39H1 methyltransferase, which establishes the H3K9me3 mark, reduced the levels of H3K9me3 in the aged mouse hippocampus [43], promoting dendrite growth and stability. It also induced glutamate receptor 1 expression in hippocampal synaptosomes, ultimately rejuvenating hippocampal memory [56].

The immediate early genes (IEGs), known as molecular markers for synaptic plasticity and long-term memory, play a role in signal transmission during memory consolidation [57]. However, in advanced age, IEGs are significantly downregulated, thus decreasing memory and learning capabilities. The reduction of IEGs might be associated with an overexpression of H3K9me3 at the IEGs promoter, which forms condensed chromatin structures and subsequently leads to a reduction in protein expression. A marked increase in SUV39H1, which leads to an H3K9me3 rise, is evident in the hippocampus of aged mice. Inhibition of SUV39H1 reduces H3K9me3 and reverses the above-mentioned hippocampal symptoms [56]. H3K9me3 levels are elevated with age in the promoter regions of key molecules that regulate synaptic plasticity. Consequently, BDNF and the IEGs such as *egr-1*, *c-fos*, and *Arc* are diminished, leading to memory deficits. By using specific inhibitors of methyltransferases SUV39H1, it has been demonstrated that synaptic plasticity and stability are enhanced and memory is restored [41]. Of importance, alterations in histone methylation associated with aging also take place in regions important for memory. Specifically, in the hippocampus of rats with memory loss due to age (between 19 and 22 months of age), there was an increase in H3K4me3 levels in comparison to their younger counterparts (at 3 months) [58]. Furthermore, it was observed that significant increases in H3K4me3 and H3K9me2 levels were evident only in younger rats undergoing novel object recognition training. This implies that impaired reactivity in histone methylation processes may play a role in the memory decline observed in aged rats. Of interest, both the deficits in learning and H3K4me3 levels were restored when the aged rats were exposed to environmental enrichment, indicating the plasticity of histone methylation [59]. Alterations in the lcRNA NEAT1 have been associated with age-related changes at H3K9me2 levels. This lcRNA increases in the hippocampus of aging mice and was shown to interact with the methyltransferase euchromatic histone lysine methyltransferase 2 (EHMT2) [60]. In nematodes, there are homologous proteins known as SET-6 and BAZ-2 involved in mitochondrial gene suppression. Interestingly, upon *Set-6* and *Baz-2* knockout, a notable increase in neural activity was detected in aged nematodes, which was further associated with the age-related decline in behavioral functions. Similarly, silencing the *Baz2b* gene in rats led to a postponement of age-related cognitive decline.

Furthermore, it has been confirmed both in mice and in humans that there is an increase in EHMT1 in Alzheimer’s disease. Pharmacological inhibitors such as BIX01294 were shown to counteract the increased EHMT1 levels in mice, maintain proper mitochondrial function, and improve learning and memory [47]. It is therefore evident that increased H3K9 expression takes place both in AD and in aging. If H3K9 methylation takes place within the promoter regions of genes associated with mitochondrial function, it exhibits a negative effect on memory and learning, primarily due to the impairment of mitochondrial functions [41].

## 6. Therapeutic Targeting of Histone Methylation

Given our understanding of the molecular processes that participate in brain aging, numerous therapeutic approaches, particularly those involving epigenetic regulation, have attracted significant attention and extensive investigation. Among recent developments targeting histone modifications, most strategies have been focused on altering histone acetylation levels. However, histone methylation exhibits a more predominant role, especially in the brain aging process, and has recently emerged as a growing and promising therapeutic field.

**Figure 3:**
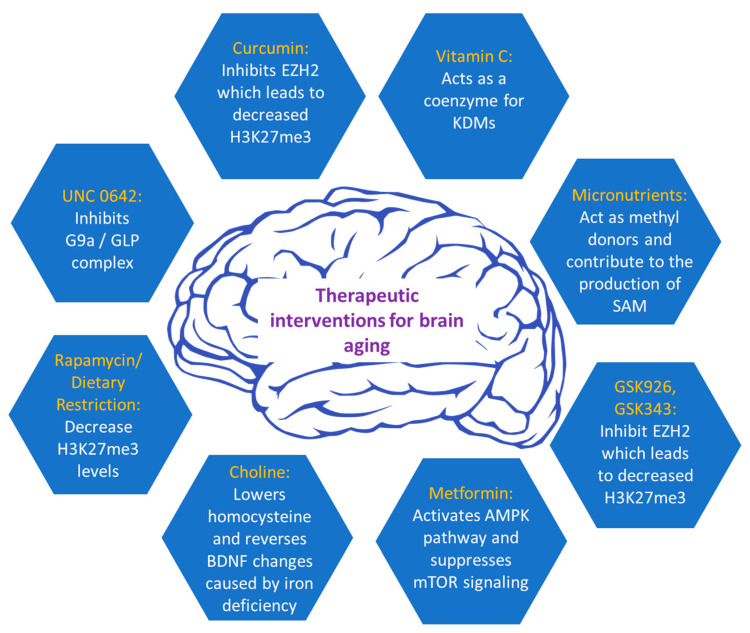
Current therapeutic interventions to alleviate brain aging.

Currently, a wide array of inhibitors is accessible for modulating histone methylation processes, targeting either methyltransferases or demethylases, and regulating their activity (Figure 3). The G9a/GLP complex, involved in the methylation of lysine residues, regulates long-term depression (LTD), long-term potentiation (LTP), and enhances BDNF expression, contributing to learning and memory [61]. Due to the growing body of evidence linking the G9a/GLP complex to various human diseases, it has emerged as a promising target for pharmacological intervention. Several small-molecule compounds have been developed to inhibit these enzymes, with UNC0642 being the most studied, exhibiting an IC50 value of less than 2.5 nM and optimized pharmacokinetics [62,63]. Studies have demonstrated that UNC0642 exerts neuroprotective effects in a mouse model that has been genetically modified to simulate early-onset Alzheimer’s disease (EOAD). This inhibition was shown to improve cognitive performance by reducing repressive chromatin marks like H3K9me2 and altering 5-mC and 5-hydroxymethylcytosine (5-hmC) levels. Notably, UNC0642 prevents the accumulation of Aβ plaques, enhances synaptic plasticity, and restores neuronal markers that are typically lost in AD. Additionally, UNC0642 reduces oxidative stress and neuroinflammation. Therefore, these findings suggest that a promising therapeutic strategy for AD may be the inhibition of G9a/GLP activity [64].

The repressive histone methyltransferase EZH2 is important in regulating cell proliferation and determining the neuronal lineage of adult neural stem cells, located in the subventricular zone (SVZ). Moreover, EZH2 is essential for the progenitors’ cell proliferation and their subsequent differentiation into neurons in the subgranular zone (SGZ) of adult mice [65]. Upon loss of EZH2, a reduction in neurogenesis was observed in SVZ NSCs both in laboratory settings (in vitro) and within living organisms (in vivo). EZH2 was shown to be directly recruited to the *Ink4a/Arf* and *Olig2* genes, facilitating H3K27 methylation and leading to suppression of gene expression. This repression of *Ink4a/Arf*, and *Olig2* was necessary for the proliferation of SVZ neurogenesis. Collectively, EZH2 has emerged as a pivotal epigenetic regulator, exerting its influence on neurogenesis during both embryonic and adult stages in both the SVZ and SGZ regions [65]. Since the first FDA approval in 2012, several potent and highly selective inhibitors have been developed that compete with SAM in inhibiting EZH2 methyltransferase activity. Most of these inhibitors have a 2-pyridone core, which partially occupies the binding pocket of EZH2 for the methyl donor (SAM) [66]. Furthermore, other inhibitors that compete with SAM for EZH2 have been developed, such as GSK343 and GSK926 [62]. GSK926 and GSK343 offer promising therapeutic potential in brain aging by reducing histone H3K27me3 levels and inhibiting EZH2 activity [67]. Targeted inhibition of the main molecular pathways may hold the key to addressing age-related cognitive decline and promoting brain rejuvenation.

Furthermore, metformin, a drug known to reduce the major pathological events and symptoms of diabetes, has been shown to delay the aging process in *C. elegans* by influencing the balance of S-adenosylmethionine and S-adenosylhomocysteine, potentially impacting histone methylation [68]. Metformin was shown to increase DNA methylation of the *Bdnf* gene in a spatial restraint stress murine model and exhibited anti-depressant effects [69]. Additionally, it can increase DICER1 levels, which is a microRNA-processing protein that regulates microRNAs involved in senescence and aging [70]. Moreover, metformin has demonstrated its efficacy in alleviating various age-related diseases in rodent animal models, including cognitive decline and neurodegeneration, as well as age-related developmental and metabolic characteristics [71]. The neuroprotective effects of metformin have been associated with its ability to induce autophagy [72]. This mechanism involves the activation of 5′ AMP-activated protein kinase (AMPK) and the suppression of mTOR signaling, leading to enhanced autophagic processes and facilitating the lysosomal degradation of Aβ [73]. As a result, metformin significantly reduces Aβ levels in the brain. Recent findings indicate that metformin’s activation of AMPK plays a role in preventing the dopaminergic neurons’ degeneration in the substantia nigra [74]. Furthermore, cell-based studies have shown that metformin protects PC12 cells and hippocampal neurons from oxidative damage induced by H_2_O_2_ by activating the AMPK pathway. It also mitigates cell death induced by cadmium (Cd) exposure in PC12 cells, SH-SY5Y cells, and primary neurons by reducing abnormal ROS accumulation [75]. These findings suggest that metformin may have a substantial neuroprotective role by targeting key aspects of brain aging.

Regarding nutraceuticals, rapamycin, a compound originally derived from soil samples collected on Easter Island, has been shown to attenuate DNA methylation changes in the hippocampus and affect brain aging [76]. In addition, rapamycin shares common effects with dietary restriction on several aging-related histone modifications, such as H3K18ac, H3K4me2, and specifically H3K27me3. Both rapamycin and dietary restriction lead to a significant decrease in the H3K27me3 mark, which, at high levels, is directly linked to brain-aging-related diseases. This evidence indicates that these histone modifications may have a dual role in regulating both aging and interventions aimed at mitigating the aging process [77]. Dietary (caloric) restriction (DR) is a well-established approach that delays aging and enhances resistance to diseases, a phenomenon conserved across various species from yeast to primates, including humans [78]. DR has been shown to ameliorate aging-related epigenetic changes such as DNA methylation and histone modifications [79,80]. It can induce neuroprotective properties and thus, mitigate cognitive age-related decline by enhancing synaptic plasticity, as demonstrated by increased long-term potentiation (LTP) [81]. Notably, brain aging has been counteracted in senescence-accelerated OXYS rats with rapamycin treatment [82].

Moreover, the one-carbon metabolism presents a vital metabolic pathway employed for the generation of methyl donors from dietary nutrients, indicating that a person’s health can be heavily influenced by dietary choices. One carbon metabolism pathway produces adequate amounts of SAM, which serves as the substrate for most methylation reactions in mammals [83]. The availability of micronutrients such as folate, betaine, methionine, vitamin B6, B2, and B12, and choline, which act as methyl donors, affects the rate of SAM production [84,85]. In one carbon metabolism, most methyl groups are derived from choline (60%), 20% from methionine, and 10–20% from folate, indicating that these micronutrients exert a neuroprotective role in mammals [86].

Vitamin C holds a crucial role as an antioxidant molecule within the brain. In conditions with a normal pH level, vitamin C primarily exists in the form of ascorbate anion [87]. Ascorbate functions as a coenzyme for methyl-cytosine dioxygenases, enzymes responsible for the process of DNA demethylation. It also serves as a potential coenzyme for specific histone demethylases of the Jumonji C domain-containing family, which catalyze the removal of methyl groups from histones. A deficiency in vitamin C might contribute to neurocognitive dysfunction and impairment, and there is evidence suggesting that ascorbic acid could have therapeutic benefits in addressing age-related diseases, including neurodegenerative disorders [88]. In tissue-specific stem cell cultures, the inhibition of demethylase activity was associated with H3K9me2/me3, such as KDM3A (JMJD1A), KDM4C (JMJD2C), and KDM7C (PHF2), resulting in the early onset of senescence, heightening DNA damage, and genomic instability. Therefore, vitamin C could be pivotal in addressing genomic instability associated with aging and fostering rejuvenation in the brain.

Curcumin is a phenolic compound isolated from the herb *Curcuma longa*, commonly known as turmeric, and is the predominant curcuminoid in this plant. It possesses anti-inflammatory, antioxidant, and anticancer characteristics and serves as a potent modulator of the epigenetic landscape [89]. It can suppress DNMTs expression and is involved in the modulation of methylation, acetylation, ubiquitination, and phosphorylation of histones, along with the regulation of miRNAs (microRNAs) [90]. Specifically, curcumin is reported to be an EZH2 inhibitor. EZH2 is a methyltransferase for the H3K27me3 mark. As stated before, there is a clear increase in this mark during aging, causing a wide array of dysregulation in neuronic functions. Consequently, the inhibition of this methyltransferase, which decreases H3K27me3 levels, suggests that curcumin may be a promising therapeutic intervention for brain aging.

Choline is a methyl donor involved in the folate-mediated one-carbon metabolic pathway generating S-adenosylmethionine [80], which provides methyl groups to DNA methyltransferases and HMTs [91]. Numerous studies have shown that cerebral choline variations are linked to alterations in the epigenetic markers of essential genes related to cognition [92]. Choline nutrition in early life is of major importance because it can affect brain development and exhibit enduring effects on brain function through epigenetic processes. Consequently, choline emerges as a neuroprotective micronutrient capable of enhancing cognitive functions throughout an individual’s lifespan. It is evident that choline supplements during the prenatal or perinatal period have been shown to enhance memory-related task performance during aging [93]. Furthermore, with regard to modifications in histone methylation, a lack of iron during the fetal and neonatal phases was shown to induce modifications to the methylation pattern of the promoter region of *Bdnf* in the rat hippocampus. Increased repressive histone marks like H3K9me3 but reduced activating histone marks such as H3K4me3 were observed in the *Bdnf* gene, resulting in decreased BDNF expression. However, the epigenetic changes in the *Bdnf* gene that were observed in iron-deficient rats during gestation were reversed upon administration of choline supplementation [94]. Furthermore, up to date, several studies have shown the durable influence of choline on brain function across generations of mice with AD [95]. Supplementing choline in a mouse with AD improved cognitive functions in both the initial and subsequent generations of mice. Additionally, it lowered homocysteine in the brain, a factor that has shown a positive correlation with an elevated risk of developing AD [85].

Although various agents and interventions targeting brain aging show satisfactory results in animal models, their effects in humans are still under investigation to estimate safety concerns and result in inconsistency. Several clinical trials exploring the role of diet restriction and exercise, as well as metformin’s and rapamycin’s effects on epigenetic aging, MCI, and neurodegenerative diseases, are still ongoing and summarized in Table 2. Currently, only metformin administration has been shown to improve cognitive impairment in a pilot randomized placebo-controlled clinical trial [96]. Of note, an interventional clinical trial focused on reversing epigenetic and other markers of senescence through the transfusion of young plasma to older human subjects (NCT03353597) is also in progress and is expected to provide important insights into the aging process.

## 7. Conclusions and Future Perspectives

In this comprehensive review, we have delved into the intricate relationship between histone methylation and the shaping of brain aging. Through the exploration of various studies, it is evident the dynamic nature and multifaceted function of histone methylation in controlling gene expression, chromatin structure, and cellular functions within the aging brain. The maintenance of neural stem cell activity, synaptic plasticity, and cognitive function rely on the precise regulation of histone methylation patterns. Dysregulation of these patterns, whether through the aberrant activation or repression of specific methyltransferases or demethylases, can lead to a cascade of events that ultimately contribute to age-related cognitive decline and neurodegenerative disorders.

One striking aspect of histone methylation in brain aging is its plasticity. Several studies have shown that environmental factors, including diet, exercise, and stress, can influence histone methylation patterns, offering a potential avenue for interventions aimed at promoting healthy brain aging. Understanding the mechanisms underlying these epigenetic changes and their interactions with genetic and environmental factors is essential for the discovery of therapeutic approaches.

It is evident that further research is imperative to elucidate the intricate network of epigenetic interactions governing the process of brain aging. The pharmaceutical compound UNC0042 has displayed promising outcomes in rodent models, specifically in the context of AD, and merits extensive investigation to explore its potential as a therapeutic intervention for AD in humans. Additionally, in-depth in vivo studies on EZH2 are required, given its role in the regulation of neurogenesis, which could have significant implications for combating brain aging. Metformin is also recognized for its neuroprotective properties and offers promise as a drug promoting brain health. However, rigorous research remains essential to fully comprehend its therapeutic potential.

While further research is needed for the above-mentioned drugs to be approved for public use, a proactive approach to mitigate the effects of brain aging and foster cognitive well-being, involves adopting a brain-enhancing dietary regimen. Mindful eating and portion control are integral aspects of a brain-healthy diet. Implementing practices such as intermittent fasting and calorie restriction can have anti-aging effects on the brain, attributed to their influence on histone modifications. It is of paramount importance to incorporate foods rich in micronutrients critical for one-carbon metabolism, encompassing choline, folate, methionine, betaine, vitamins B2, B6, and B12, as they are essential for the generation of S-adenosylmethionine, the pivotal substrate in methylation reactions throughout the body. Additionally, antioxidants and anti-inflammatory agents found in foods like curcumin, citrus fruits, and berries (abundant in vitamin C) are vital for brain health. Complementing these anti-oxidant and anti-inflammatory agents, dietary sources such as eggs, meat, fish (sources of choline), and leafy greens (rich in folate) should constitute integral components of one’s daily nutritional intake.

As we move forward, the concept of personalized medicine in the context of brain aging should not be overlooked. Genetic and epigenetic diversity among individuals necessitates tailored approaches to therapy and prevention. However, the implementation of personalized medicine in the field of epigenetics raises ethical concerns related to privacy, consent, and access to healthcare. Striking a balance between personalized treatments and ethical considerations will be a challenge that researchers and policymakers must address. Hence, concerted efforts should be undertaken to ensure the accessibility of pharmaceuticals, including personalized medications, to individuals across diverse socioeconomic strata. Brain aging constitutes a global challenge, and as such, its remediation should be universally attainable. Furthermore, it is imperative that these pharmaceutical solutions not only be readily available but also tailored for the convenience of patients. Formulations, whether in the form of oral tablets or injectable preparations, must be meticulously crafted to facilitate patients’ seamless integration of treatment into their daily routines without imposing limitations on their regular activities.

Moreover, the study of epigenetic changes in glial cells, neurons, and neural stem cells is essential for a comprehensive understanding of brain aging. Each cell type may exhibit unique epigenetic profiles and responses to aging-related stressors, and deciphering these differences could provide valuable insights into the heterogeneity of age-related neurodegenerative disorders. The integration of omics approaches, such as genomics, transcriptomics, and proteomics, with epigenetics will be instrumental in advancing our understanding of brain aging. High-throughput technologies can allow researchers to identify molecular alterations occurring during aging, which will allow us to identify key pathways and molecular players that can be targeted for intervention. This in turn will enable the development of targeted therapies or interventions to slow down or even reverse some epigenetic changes and, in this way, enhance the future treatment of age-related neurological diseases and conditions. In addition, an in-depth understanding of the molecular mechanisms often helps with the early detection and even prevention of a disease. Early detection can lead to better management and treatment outcomes, facilitating the development of preventive strategies to delay or reduce the impact of brain aging. This combination will give the patient the opportunity to receive personalized medical treatment, which can potentially reduce the economic burden on healthcare systems and families. By understanding an individual’s specific molecular risk factors for age-related brain diseases, healthcare providers can tailor treatments and interventions to suit their unique needs. Altogether, slowing down or mitigating the effects of brain aging can help people maintain cognitive function, memory, and independence as they grow older, thereby enhancing their overall quality of life.

Apart from the individual benefits, investigating brain aging might result in the identification of novel targets for drug development. Pharmaceuticals have the potential to create medications that address the root factors responsible for brain aging, potentially leading to more effective treatments for a wide range of neurological conditions, thus contributing to our broader understanding of neurobiology and the aging process.

In conclusion, the relationship between histone methylation and brain aging is a complex and dynamic field that holds great promise for understanding and ultimately mitigating age-related cognitive decline and neurodegenerative diseases. The plasticity of histone methylation patterns, the potential for targeted interventions, and the integration of omics approaches offer exciting avenues for future research. However, the road to translating these discoveries into effective therapies is fraught with challenges, requiring interdisciplinary collaboration, rigorous ethical considerations, and a clearer insight into epigenetic regulation’s molecular intricacies involved in aging brains. The journey ahead is arduous, but the potential benefits for the aging population are immeasurable, making this a field of paramount importance in the years to come.

## Figures and Tables

**Table 2:** Ongoing clinical trials related to epigenetic targets and brain aging.

Drug/Intervention	Condition/Disease	Trial No.	Phase
Diet	Epigenetic aging	NCT04962464, NCT05297097	2 2
Exercise	Aging; Alzheimer disease	NCT04299308	
Metformin	Mild cognitive impairment	NCT04098666	2/3
Rapamycin	Mild cognitive impairment; Alzheimer disease	NCT04629495, NCT04200911	2 1

## Data Availability

Not applicable.
